# Characterization of a *vraG* Mutant in a Genetically Stable *Staphylococcus aureus* Small-Colony Variant and Preliminary Assessment for Use as a Live-Attenuated Vaccine against Intrammamary Infections

**DOI:** 10.1371/journal.pone.0166621

**Published:** 2016-11-17

**Authors:** Julie Côté-Gravel, Eric Brouillette, Nataša Obradović, Céline Ster, Brian G. Talbot, François Malouin

**Affiliations:** Centre d’Étude et de Valorisation de la Diversité Microbienne (CEVDM), Département de biologie, Faculté des sciences, Université de Sherbrooke, Sherbrooke, QC, Canada; University of South Dakota, UNITED STATES

## Abstract

*Staphylococcus aureus* is a leading cause of bovine intramammary infections (IMIs) that can evolve into difficult-to-treat chronic mastitis. To date, no vaccine formulation has shown high protective efficacy against *S*. *aureus* IMI, partly because this bacterium can efficiently evade the immune system. For instance, *S*. *aureus* small colony variants (SCVs) have intracellular abilities and can persist without producing invasive infections. As a first step towards the development of a live vaccine, this study describes the elaboration of a novel attenuated mutant of *S*. *aureus* taking advantage of the SCV phenotype. A genetically stable SCV was created through the deletion of the *hemB* gene, impairing its ability to adapt and revert to the invasive phenotype. Further attenuation was obtained through inactivation of gene *vraG* (SACOL0720) which we previously showed to be important for full virulence during bovine IMIs. After infection of bovine mammary epithelial cells (MAC-T), the double mutant (Δ*vraG*Δ*hemB*) was less internalized and caused less cell destruction than that seen with Δ*hemB* and Δ*vraG*, respectively. In a murine IMI model, the Δ*vraG*Δ*hemB* mutant was strongly attenuated, with a reduction of viable counts of up to 5-log10 CFU/g of mammary gland when compared to the parental strain. A complete clearance of Δ*vraG*Δ*hemB* from glands was observed whereas mortality rapidly (48h) occurred with the wild-type strain. Immunization of mice using subcutaneous injections of live Δ*vraG*Δ*hemB* raised a strong immune response as judged by the high total IgG titers measured against bacterial cell extracts and by the high IgG2a/IgG1 ratio observed against the IsdH protein. Also, Δ*vraG*Δ*hemB* had sufficient common features with bovine mastitis strains so that the antibody response also strongly recognized strains from a variety of mastitis associated *spa* types. This double mutant could serve as a live-attenuated component in vaccines to improve cell-mediated immune responses against *S*. *aureus* IMIs.

## Introduction

*Staphylococcus aureus* is a major human and animal pathogen that can cause high morbidity, acute infections, as well as difficult-to-treat chronic forms of diseases. Among factors that can explain the failure of antibiotherapy and the tendency to cause chronic infections, many have noted the pathogen’s multifaceted virulence, predominantly its abilities to impair or elude host immune responses by toxin secretion [1,2], formation of biofilm [3] and survival in non-phagocytic host cells, which may shield the pathogen from the action of host immune system and antibiotics [4]. Furthermore, incidences of *S*. *aureus* infections are becoming more worrisome with the emergence of multiple antibiotic resistant strains [5,6]. Consequently, there is an urgent need to find potent new strategies to control this pathogen.

As for today, bovine mastitis is still an important problem for the dairy industry, and *S*. *aureus* is the most frequent pathogen in all combined cases of clinical and subclinical intramammary infections (IMIs) [7]. Subclinical IMIs in particular can be a real concern: they often stay unnoticed by producers, are highly transmissible during milking and thus result in chronic infections that can persist for the life of the animal [8]. Over time, they can generate tissue damage that rapidly leads to a decrease in milk production and quality [9].

The development of vaccines for the prevention and control of *S*. *aureus* IMIs has been extensively investigated, although no formulation has demonstrated high protective efficacy to date. According to several reviews of the different commercially available and experimental vaccine formulations, this lack of protection is possibly caused by inadequate vaccine targets [10,11], high diversity among strains capable of provoking mastitis [10,12,13] or the failure to elicit an appropriate immune response [14–16]. It is increasingly understood that immunity solely based on vaccine-induced antibodies may be important, but is however insufficient for inducing protection against *S*. *aureus* [10,11]. It appears that cell mediated immunity (CMI) based on Th1 and Th17 type responses may be necessary to complete the protection [15–18].

In a previous study, we used a DNA microarray approach to uncover *S*. *aureus* genes that were highly expressed during bovine IMIs [19]. One gene (*guaA*) was shown to be a good target for a new drug therapy [20], and other genes were further investigated as vaccine candidates. Gene *vraG* (SACOL0720) was shown to be likely induced by the growth of *S*. *aureus* in fresh milk both *in vitro* and *in vivo*. The importance of gene *vraG* in *S*. *aureus* virulence was also demonstrated by the significant attenuation of growth observed for the gene inactivation mutant during bovine IMI [19].

It is now recognized that *S*. *aureus* small colony variants (SCVs) add important contributions to chronic infections and therapy failures. This may be attributed to the particular features of SCVs that make this phenotype adapted for long-term persistence in host tissues via expression of a distinct set of virulence factors [21], and that also allow survival in host cells [22,23]. Since SCVs have an improved ability for internalization into cells [4,24,25] and can colonize the host without generating invasive infections or tissue destruction [26,27], we hypothesized that these features could be of value in the development of genetically attenuated *S*. *aureus* strains. The use of *S*. *aureus* live-attenuated bacteria as vaccines represents an interesting approach to improve immune responses. Live-attenuated organisms that mimic natural infections stimulate the immune system in a powerful manner, eliciting broad and robust immune responses that increase serum and mucosal antibodies as well as effector and memory T cells which act synergistically to protect against disease [28,29].

In this study, we generated a *vraG* mutation in a SCV background to create an attenuated strain for vaccine purposes. Inactivation of gene *vraG*, should prevent cationic peptide resistance [30–32] and reduce virulence [19], while inactivation of gene *hemB* creates a stable SCV and prevents reversion to the invasive phenotype, a phenomenon normally seen during *S*. *aureus* infections [33]. We evaluated the persistence of the double mutant in a bovine mammary epithelial cells and demonstrated its attenuation and safety in a murine IMI model. We also report some immunogenic properties of this vaccine strain. This work is a first step in the proof of concept needed for the development of a live-attenuated vaccine for immunization and protection against *S*. *aureus* IMIs.

## Materials and Methods

### Ethics statement

The animal experiments were conducted following the guidelines of the Canadian Council on Animal Care and the institutional ethics committee on animal experimentation of the Faculté des Sciences of Université de Sherbrooke. The institutional ethics committee on animal experimentation of the Faculté des Sciences of Université de Sherbrooke specifically approved this study.

### Bacterial strains and growth conditions

Strains used in this study are listed in Table 1. *S*. *aureus* ATCC 29213 and its isogenic mutant Δ720 were previously described [19]. Strain Δ720 is an intron insertion mutant of gene *vraG* that was renamed in this study Δ*vraG* for clarity. For the immunological tests, we selected four different bovine mastitis isolates corresponding to some of the predominant *S*. *aureus spa* types found in Canadian dairy herds and elsewhere in the world [13,34]. Strain SHY97-3906 (*spa* t529) was isolated from a case of clinical bovine mastitis that occurred during the lactation period, and CLJ08-3 (*spa* t359) was originally isolated from a cow with persistent mastitis at dry-off [19]. Strains Sa3151 (*spa* t13401) and Sa3181 (*spa* t267) were obtained from the Canadian Bovine Mastitis and Milk Quality Research Network (CBMMQRN) Mastitis Pathogen Culture Collection, and were isolated from cases of subclinical IMIs. Unless otherwise stated, *S*. *aureus* strains were grown in tryptic soy broth (TSB) and agar (TSA) (BD, Mississauga, ON, Canada), and *Escherichia coli* DH5α was grown in LB and LBA medium (BD). The ability of *S*. *aureus* strains to produce biofilm *in vitro* was evaluated as described before [13]. Whenever required, ampicillin (100μg/ml) (Sigma-Aldrich, Oakville, ON, Canada), chloramphenicol (20 μg/ml) (ICN Biomedicals, Irvine, CA), and erythromycin (10 μg/ml) (Sigma) were added to culture media.

**Table 1:** Strains and plasmids used in this study.

Strain or plasmid	Relevant details	Source or Reference
**Strains**		
***S*. *aureus***		
**RN4220**	Derivative of 8325–4, Restriction-deficient strain that accepts DNA from *E*. *coli*	[35]
**ATCC 29213**	Wild-type, VraG positive, normal phenotype	ATCC 29213
**Δ*vraG***	*vraG* (SACOL0720) intron insertion mutant, isogenic to ATCC29213	[19]
**Δ*hemB***	*hemB*::Em^r^; isogenic mutant of ATCC29213, SCV phenotype	This study
**Δ*vraG*Δ*hemB***	*hemB*::Em^r^; isogenic mutant of Δ*vraG* SCV phenotype	This study
**SHY97-3906**	Isolate from a dairy cow with a case of clinical mastitis occurring during the lactation period; *spa* type t529	[19]
**CLJ08-3**	Isolate from a dairy cow with a case of subclinical IMI persisting through the dry-off period; *spa* type t359	[19]
**Sa3151**	Isolate from a dairy cow subclinical IMI occurring during the lactation period; *spa* type t13401	This study
**Sa3181**	Isolate from a dairy cow subclinical IMI occurring during the lactation period; *spa* type t267	This study
***E*. *coli***		
**DH5α**	F– Φ80*lac*ZΔM15 Δ(*lac*ZYA-*arg*F) U169 *rec*A1 *end*A1 *hsd*R17 (rK–, mK+) *pho*A *sup*E44 λ– *thi*-1 *gyr*A96 *rel*A1	Invitrogen Life Technologies
**Plasmids**		
**pBT2**	Shuttle vector, temperature-sensitive; Ap^r^Cm^r^	[36]
**pBT-E**	pBT2 derivative, inserted *ermA* cassette; Ap^r^Cm^r^Em^r^	This study
**pBT-E*hemB***	pBT2 and pBT-E derivative, for *hemB* deletion: insertion of ~1000 bp of *hemB* flanking regions on both sides of *ErmA*; Ap^r^Cm^r^Em^r^	This study

### DNA manipulations

Recommendations from the manufacturers of kits were followed for genomic DNA isolation (Sigma), plasmid DNA isolation (Qiagen, ON, Canada), extraction of DNA fragments from agarose gels (Qiagen) and purification of PCR products and of digested DNA fragments (Qiagen). An additional treatment of 1h with lysostaphin (Sigma) at 200 μg/ml was used to achieve efficient lysis of *S*. *aureus* cells in genomic and plasmid DNA isolations. Primers were designed to add restriction sites upstream and downstream of the amplified products. PCRs were performed using the Taq DNA Polymerase (NEB, Pickering, ON, Canada) for routine PCR or the Q5 high fidelity DNA Polymerase (NEB) for cloning, and cycling times and temperatures were optimized for each primer pair. Plasmid constructs were generated using *E*. *coli* DH5α (Invitrogen, Burlington, ON, Canada), restriction enzymes (NEB), and the T4 DNA ligase (NEB). Plasmid constructs were validated by restriction digestion patterns and DNA sequencing before electroporation in *S*. *aureus* RN4220 [35] and in final host strains. Plasmids used in this study are listed in Table 1.

### Generation of pBT-E:*hemB* and insertional deletion of *hemB*

Isogenic *hemB* mutants of the ATCC 29213 and Δ*vraG* strains were constructed, in which the *hemB* gene was deleted and replaced by the insertion of an *ermA* cassette by homologous recombination. The temperature-sensitive [36] pBT2-*hemB*:*ermA* (pBT-E:*hemB*) was used in a strategy previously described [37], with some modifications. Briefly, the pBT-E plasmid was constructed by the insertion of an *ermA* cassette between the *Xba*I and *Sal*I sites of the temperature-sensitive shuttle vector pBT2. The flanking regions of gene *hemB* [38] DNA fragments were amplified from *S*. *aureus* ATCC 29213 and were cloned on both sides of the *ermA* cassette into the plasmid pBT-E. The plasmid was then transferred for propagation into *S*. *aureus* RN4220 (res-). After bacterial lysis with lysostaphin (200 μg/ml for 1 h at room temperature), plasmid DNA was isolated and used to transform ATCC 29213 and Δ720 by electroporation. For plasmid integration and mutant generation, bacteria were first grown overnight at 30°C with 10 μg/ml of erythromycin and a 1 μg/ml hemin supplementation (Sigma). Bacteria were then diluted 1:1000 and grown overnight at 42°C with 2.5 μg/ml of erythromycin and 1 μg/ml hemin. This step was repeated twice. Finally, bacteria were diluted 1:1000 and grown overnight at 42°C without antibiotics. Mutants with the inactivated *hemB* gene were selected as resistant to erythromycin and sensitive to chloramphenicol, together with a SCV phenotype that can be complemented *(i*.*e*., reversion to the normal growth phenotype) by a 5 μg/ml hemin supplementation on agar plates. The deletion of *hemB* in the ATCC 29213 and Δ*vraG* strains was confirmed by PCR and DNA sequencing of the PCR product.

### Hemin supplementation in broth culture

To evaluate the capacity of hemin to restore optimal growth kinetics of *S*. *aureus* Δ*hemB* and the double mutant Δ*vraG*Δ*hemB*, overnight bacterial cultures were diluted to an *A*_600 nm_ of approximately 0.1 in culture tubes containing fresh BHI supplemented with hemin (Sigma) added at various concentrations. The *A*_600nm_ of cultures was monitored at different points in time during the incubation period at 35°C (225 rpm).

### *S*. *aureus* infection of bovine mammary epithelial cells

An established bovine mammary epithelial cell (BMEC) line, MAC-T, was used as a cell culture model of infection [39], and was used for the characterization of intracellular infectivity and persistence of *S*. *aureus* ATCC 29213 and its isogenic mutants. The MAC-T cells were routinely cultured and maintained in Dulbecco’s modified Eagle’s medium (DMEM) containing 10% heat-inactivated fetal bovine serum (FBS), supplemented with 5μg/ml insulin (Roche Diagnostics Inc., Laval, QC, Canada) and 1μg/ml hydrocortisone (Sigma), and incubated at 37°C in a humidified incubator with 5% CO_2_. Cell culture reagents were purchased from Wisent (St-Bruno, QC, Canada).

Forty-eight hours before infection, 1x10^5^ MAC-T cells per ml were seeded on treated CellBIND^®^ 24-well plates (Corning) to obtain 30% confluence. Monolayers were grown to confluence under 5% CO_2_ at 37°C. Six hours prior to infection, monolayers were washed with DMEM and incubated with an invasion medium (IM) (growth medium without antibiotics containing 1% heat-inactivated FBS). Overnight bacterial cultures were diluted 1:20 in fresh TSB and grown to mid-logarithmic growth phase, then washed with PBS and diluted in IM to a multiplicity of infection of 10. Invasion was achieved by incubating monolayers with bacteria for 3 h. Monolayers were then washed with DMEM and incubated with IM containing 20 μg/ml lysostaphin to kill extracellular bacteria. The use of lysostaphin to kill extracellular normal and SCV *S*. *aureus* was previously validated in cell invasion assays [24,39]. The treatment was allowed for 30 min before the determination of intracellular CFUs after 3h of infection, or the treatment was extended for an additional 12 or 24 h for those later time points. For CFU determination, following extensive washing with Dulbecco's Phosphate-Buffered Saline (DPBS), monolayers were detached with trypsinization and lysed with 0.05% Triton X-100 before PBS was added to obtain a final 1X concentration. The lysate was serially diluted and plated on TSA for CFUs determination.

### BMECs viability and metabolic activity assay

To determine the cytotoxic damage inflicted by *S*. *aureus* ATCC 29213 and its isogenic mutants on MAC-T cells, a cell metabolic activity assay that measures the reduction of 3-[4,5-dimethylthiazol-2-yl]-2,5 diphenyl tetrazolium bromide (MTT) into an insoluble formazan product in viable cells, was performed. The assay followed the method of Kubica *et al*. [40] with some modifications. Briefly, *S*. *aureus* infection of cells was achieved as described in the persistence assay, but instead of inducing cell lysis after 12 h or 24 h, cells were incubated with 100 μl of the MTT reagent (5 mg/ml) (Sigma) in DPBS for 2 h at 37°C. Following this, an acidic solvent solution of 16% SDS and 40% PMF, pH 4.7, was added to lyse the cells and solubilize the crystals of formazan overnight. The samples were read using an Epoch microplate reader (Biotek Instruments Inc.) at a wavelength of 570 nm. All assays were performed in triplicate, and control wells with uninfected cells (high viability control) or lysed bacteria-infected cells (bacteria-background control; treated with 0.05% Triton X-100 for 10 min before MTT addition) were included to each plate. The level of metabolic activity was calculated using the following formula:

((Absorbance of the sample – Absorbance of bacteria − background control) / High viability control) × 100

### Virulence in the mouse mastitis model

The mouse mastitis model of infection was based on previously described work [4,41]. Briefly, one hour following removal of 12–14 day-old offspring, lactating CD-1 mice (Charles River Laboratories) were anesthetized with ketamine and xylazine at 87 and 13 mg/kg of body weight, respectively, and mammary glands were inoculated under a binocular. Mammary ducts were exposed by a small cut at the near ends of teats and a 100 μl-bacterial suspension containing ~10^2^ CFUs in endotoxin-free phosphate-buffered saline (PBS, Sigma) was injected through the teat canal using a 32-gauge blunt needle. Two glands (fourth on the right [R4] and fourth on the left [L4] from head to tail) were inoculated for each animal. Mammary glands were aseptically harvested at the indicated times, weighed and visually evaluated for inflammation. Bacterial burden was evaluated after mechanical tissue homogenization in PBS, serial dilutions, and plating on agar for CFU determination. In additional experiments, homogenized glands were preserved for protein extraction and myeloperoxidase (MPO) activity assays.

### Mammary gland protein extraction

Total protein extraction from mammary glands was performed by an optimized method previously described [42], with some modifications. Mammary tissues were homogenized in a buffer containing a final concentration of potassium phosphate of 50 mM, pH 6.0, and hexadecyltrimethylammonium bromide (CTAB) 50 mM (Sigma). The samples were then sonicated, freeze-thawed in liquid nitrogen, and centrifuged at 2000 g for 15 min at 4°C. Finally, the fat layer was removed by aspiration, and supernatants were saved for a final centrifugation of 15 min at 15 000 g, to discard all cellular debris. Supernatants were distributed in aliquots and kept at -80°C until used for the enzymatic assays or protein concentration determination as measured by the bicinchoninic acid method (BCA) Protein Assay Kit (Thermo-Scientific).

### MPO activity assay

Neutrophil recruitment in mammary tissues was measured by quantification of the MPO enzyme activity by the *o*-dianisidine-H_2_O_2_ method, modified for a microplate format [43]. In a 96-well microplate, 10 μl of tissue extraction supernatants were incubated with a solution of *o*-dianisidine hydrochloride (167 μg/ml) (Sigma) and 0.0005% H_2_O_2_ (Sigma) in 50 mM CTAB phosphate buffer 50 mM, pH 6.0. The MPO activity was measured kinetically with intervals of 15 s over a period of 5 min in an Epoch microplate reader at 460 nm. A Unit of MPO was considered as the amount of enzyme that degrades 1 μmol of H_2_O_2_/min at 25°C, assuming an absorption coefficient of 11.3 mM^−1^ cm^−1^ at 460 nm for *o*-dianisidine [44]. Results were expressed as units of MPO per g of gland.

### Mouse immunizations

The immunogenic properties of the attenuated strain Δ*vraG*Δ*hemB* administered as a live vaccine were evaluated in mice. In preliminary studies, the mice well tolerated intramuscular and subcutaneous (SC) injections of the attenuated strain. The doses of 10^6^, 10^7^ and 10^8^ CFUs and the SC route were selected for subsequent experiments. For the preparation of bacterial inoculum, *S*. *aureus* Δ*vraG*Δ*hemB* colonies previously grown on BHIA plates were washed twice in ice cold PBS and suspended in PBS containing 15% glycerol, then aliquoted and kept at -80°C until subsequent use. The viable bacterial counts in the inoculum preparation was validated by serial dilution plating on BHIA. CD-1 mice were randomly divided into 3 groups: group 1 (n = 3) received a dose of 10^6^ CFUs; group 2 (n = 3), 10^7^ CFUs, and group 3 (n = 3), 10^8^ CFUs. Mice were immunized by two subcutaneous injections of bacteria in PBS (100 μl), in the neck, two weeks apart. This live-attenuated formulation was also compared to a subunit vaccine using only the purified staphylococcal IsdH protein as the antigen. The recombinant *S*. *aureus* IsdH protein was produced in *E*. *coli* as previously described [45]; mice (n = 6) were immunized by two subcutaneous injections in the neck, three weeks apart, using 20 μg of IsdH combined to EMULSIGEN^®^-D (25% v/v) (MVP Laboratories, Inc., Omaha, NE) in a volume of 100 μl. Blood samples were taken just before the priming injection (preimmune serums) and 10–21 days after the boost immunization (immune serums). Blood aliquots were allowed to clot at room temperature for an hour and then centrifuged at 10,000 g for 10 min at 4°C. The serums were collected and kept at -20°C until subsequent analysis.

### Preparation of *S*. *aureus* cell extracts

Preparation of *S*. *aureus* whole cell extracts was done as previously described with some modifications [46]. Briefly, overnight bacterial cultures were diluted 1/1000 in fresh BHI broth, and then incubated at 35°C (225 rpm) until an absorbance value (OD_600nm_) of ~ 0.8 was reached. Bacterial cells were centrifuged and pellets were washed in ice-cold PBS twice and suspended with the addition of 5 ml of PBS per ml of pellet. Bacterial suspensions were first treated with lysostaphin (Sigma) (100 μg/ml of pellet) for 1 h at 37°C, and then 3 μg of protease inhibitor cocktail (Sigma), 8 μg of RNase A (Sigma) and 8 μg of DNase (Qiagen) per ml of pellet were added to the suspension. After 30 min at room temperature, cells were mechanically disrupted by 3 to 4 passages in a SLM Aminco French Pressure cell disrupter, and then centrifuged at 12,000 × g and 4°C for 10 min to remove unbroken cells. Supernatant was collected and total protein concentration was determined as previously described with the BCA Protein Assay Kit.

### Detection of mouse IgG by ELISA

Detection of serum total IgG against the Δ*vraG*Δ*hemB* vaccination strain and each of the bovine IMI isolates was performed to demonstrate and measure the systemic humoral response generated by the immunization of mice. For target antigens, Nunc MaxiSorpTM 96-well plates (Thermo Fisher Scientific Inc., Rochester, NY) were coated with 100 μl of each of the whole *S*. *aureus* cell extracts or of the recombinant IsdH protein (10 μg/ml diluted in carbonate/bicarbonate buffer, Sigma), and incubated overnight at room temperature. The plates were then saturated with PBS containing 5% skim milk powder for 1 h at 37°C, followed by a second blocking step with an addition of 5% porcine serum to prevent unspecific *S*. *aureus* protein A interactions, in the case of whole-cell extracts. One hundred microliters of two-fold serial dilutions of the sera in the dilution buffer (PBS with 2% milk and 0.025% TweenTM 20) were loaded into the plates and incubated for 1 h at 37°C. Plates were then washed three times with PBS containing 0.05% TweenTM 20, and loaded with 100 μl of horseradish peroxidase (HRP)-conjugated goat anti-mouse IgG, IgG1 or IgG2a (Jackson ImmunoResearch Laboratories Inc., West Grove, PA) diluted 1/5000 in the dilution buffer. After 1 h of incubation at 37°C followed by washes, peroxidase activity was detected using 3,3′,5,5′-tetramethylbenzidine (TMB) reagent (KPL Inc., Gaithersburg, MD) according to the manufacturer’s recommendations.

### Statistical analysis

Statistical analyses were carried out with the GraphPad Prism software (v.6.02). Intracellular bacterial CFUs and bacterial CFUs/g of gland (IMI in mice) were transformed in base 10 logarithm values before being used for statistical analyses. Statistical tests used for the analysis of each experiment and significance are specified in the figure legends.

## Results

### Validation of the SCV phenotype

Homologous recombination was used to generate *hemB* mutants in the *S*. *aureus* wild-type and Δ*vraG* isogenic backgrounds. The *hemB* deletion was confirmed by PCR and by sequencing of the PCR product. The gene *hemB* codes for an δ-aminolevulinate dehydratase, an essential enzyme in porphyrin biosynthesis converting δ-aminolevulnic acid to porphobilinogen [38]. Lacking this enzyme, the *hemB* mutant does not synthesize heme resulting in a defective electron transport system and ATP synthase activity. The *hemB* mutant thus produces much less energy and secondary metabolism is impaired. This phenotypically translates into a slow growth. *In vitro* characterization of mutants confirmed the expected small-colony phenotype of SCVs. After 48 h of incubation at 37°C on TSA, colonies of *S*. *aureus* Δ*hemB* and Δ*vraG*Δ*hemB* were approximately 0.5 mm in diameter and appeared non-pigmented, whereas colonies of the parent and Δ*vraG* strains were 4 mm or greater in diameter with a bright yellow pigmentation. The lack of pigmentation in SCVs was previously documented [27]. Growth of the *S*. *aureus* Δ*hemB* mutants reached a plateau at a lower bacterial density in broth culture compared to wild-type *S*. *aureus*, but chemical complementation by the addition of hemin (1 μg/ml) in TSB restored the capacity of *S*. *aureus* Δ*hemB* to reach a bacterial density equivalent to that of the parent strain (data not shown). Similar results were obtained for the Δ*vraG*Δ*hemB* double mutant compared to its isogenic strain Δ*vraG*. Wild-type and Δ*vraG* showed no difference in growth in broth cultures using TSB or milk as cultivation medium, as shown in a previous study [19]. Finally, the ATCC 29213 strain, the single mutants or the double mutant produced equivalent amounts of biofilm compared to that measured for the majority of bovine mastitis isolates studied in a previous study [13].

These results show validation of the SCV phenotypes in *hemB* mutants and demonstrate that chemical complementation by supplemental hemin restores the wild-type phenotype to the full extent.

### A mutation in gene *vraG* impairs *S*. *aureus* internalization in BMECs

**Fig 1:**
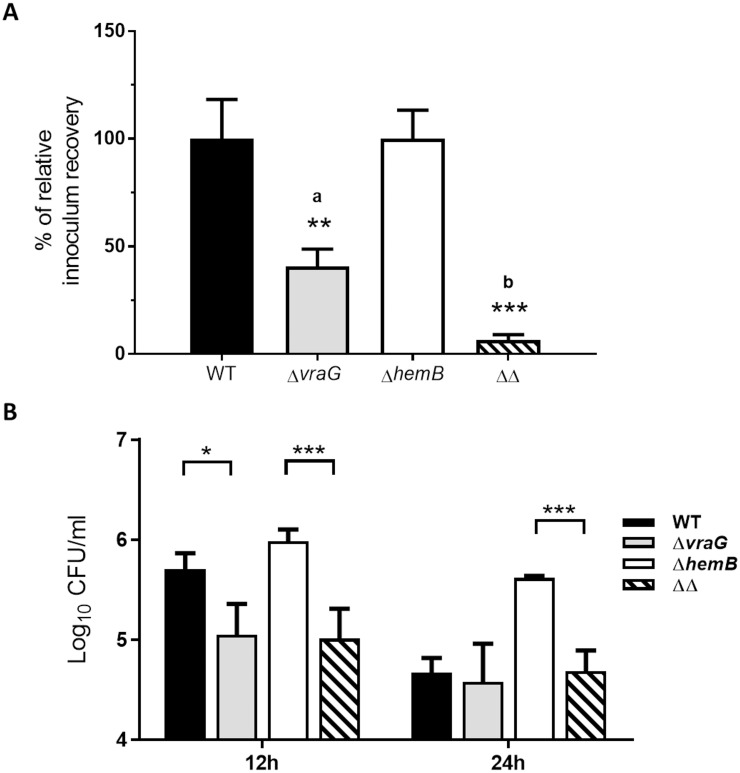
Influence of *S*. *aureus* Δ*hemB*, Δ*vraG*, and Δ*vraG*Δ*hemB* mutations on MAC-T cell infectivity. MAC-T cells were infected with each of the four strains for 3h, then were incubated with lysostaphin an additional 30 min (t = 3h), 12h or 24h and lysed for measurement of intracellular bacteria (CFUs). (A) Relative recovery of the initial inoculum found within cells at 3h for the Δ*vraG* and Δ*vraG*Δ*hemB* (ΔΔ) mutants. Results are normalized according to that obtained with ATCC 29213 (WT) for comparison to Δ*vraG*, or with Δ*hemB* for comparison to Δ*vraG*Δ*hemB* (ΔΔ), and are expressed as means with SD (**, P ≤ 0.01; ***, P ≤ 0.001; unpaired t test). (B) Means and SD of intracellular CFUs for WT and mutants at 12h (left) and 24h (right). A two-way ANOVA and Tukey's multiple comparisons test was used (*: P ≤ 0.05; ***: P ≤ 0.001). All values indicate the mean of three independent experiments, each performed in triplicate.

**Fig 2:**
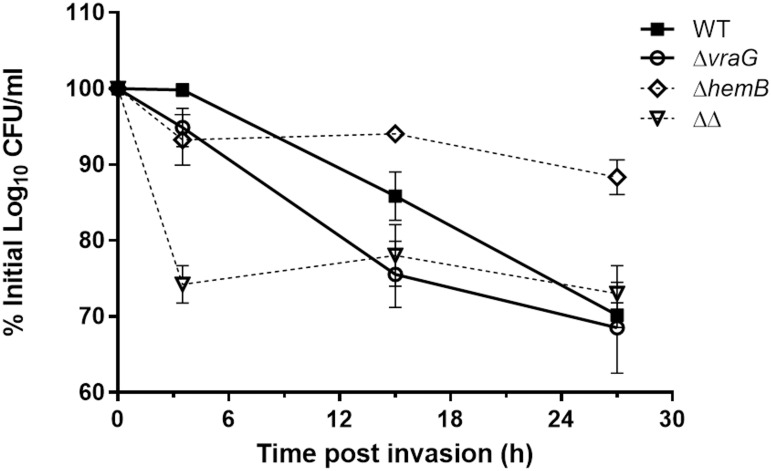
Persistence of *S*. *aureus* ATCC 29213 (WT) and isogenic mutants within MAC-T cells over time. MAC-T cells were infected with each of the four strains for 3h, then were incubated with lysostaphin an additional 30 min, 12h or 24h and lysed for measurement of intracellular bacteria (CFU). Intracellular bacterial CFUs are expressed as the percentage of the initial inoculum after being transformed in base 10 logarithmic values (Log_10_ CFU/ml). The full lines relate to strains of the normal phenotypes (WT and Δ*vraG*), whereas the dotted lines represent the strains having the SCV phenotype (Δ*hemB* and Δ*vraG*Δ*hemB* [ΔΔ]). Values indicate the mean of three independent experiments, each done in triplicate, with standard deviations.

We compared the infectivity of the wild-type, Δ*vraG*, Δ*hemB* and Δ*vraG*Δ*hemB* strains in infection and persistence assays using MAC-T cells. By comparing the three mutant strains to their isogenic parent, distinct effects of mutations in gene *hemB* and *vraG* were observed. A short 3-h incubation of bacteria with cell monolayers followed by the addition of lysostaphin to eliminate extracellular bacteria demonstrated good levels of internalization into MAC-T cells for both the wild-type and Δ*hemB* strains, based on the recovery of intracellular CFUs. On the other hand, the single Δ*vraG* mutant showed significantly less (P ≤ 0.01) internalization compared to its parental strain (Fig 1A). The reduction in internalization as seen with Δ*vraG* was even more pronounced when comparing the double mutant Δ*vraG*Δ*hemB* to Δ*hemB*, with a 10-fold reduction of inoculum recovery in the 3-h internalization assay (P ≤ 0.001, Fig 1A). This initial reduction of internalized bacterial load was still apparent 12 and 24 h post invasion (PI) for the double mutant strain Δ*vraG*Δ*hemB* (Fig 1B), as illustrated by the 1-log10 reduction of CFU/ml at both time points compared to that observed for Δ*hemB* (P ≤ 0.001). The difference in initial intracellular bacterial loads between the single Δ*vraG* mutant and wild-type strains (Fig 1A) gradually vanished with longer incubation times (Fig 1B), as both strains did not well persist in MAC-T cells (Fig 2). On the contrary, intracellular CFUs recovered for the single Δ*hemB* strain was significantly higher compared to that recovered for the three other strains at 24 h PI (Fig 1B, P ≤ 0.001 against all). Globally and as expected for the SCV phenotype, the Δ*hemB* strain showed a better intracellular persistence than any of the other strains over time (Fig 2).

These results suggest that the Δ*vraG* mutation greatly reduces the internalization process into MAC-T cells. Results further demonstrate that the Δ*vraG*Δ*hemB* mutant is still capable of internalization and persistence into BMECs, but to a lesser degree than that seen with the single Δ*hemB* mutant.

### Δ*vraG*Δ*hemB* and Δ*hemB* SCVs cause low BMEC disruption

As reported above, SCV strains showed a greater persistence over time in MAC-T cells, as illustrated by their sustained intracellular viability at 12 and 24 h PI in comparison to the wild-type and Δ*vraG* strains (Figs 1B and 2). Percent of inoculum recovered from cells stayed nearly the same from 0 to 24 h after lysostaphin addition, both for the double and single *hemB* mutants, with a slight increase at 12 h, indicating intracellular growth (Fig 2). Both strains started to decrease at a slow rate after this time point of 12 h. However, the apparent reduction of intracellular CFUs for the WT and Δ*vraG* strains was concomitant with the visual observation of increasing damage to cell monolayers over time, in comparison to that observed with strains of the SCV phenotype. This prompted us to evaluate MAC-T cells viability following infection by each of the four strains studied. MAC-T cell viability was evaluated by the MTT method in the exact same conditions that were used for the determination of intracellular bacterial counts.

**Fig 3:**
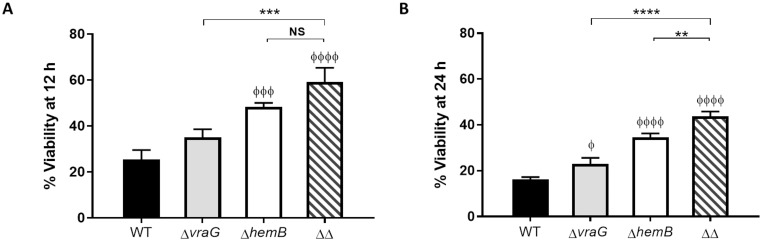
Viability of MAC-T cells infected by *S*. *aureus* ATCC 29213 (WT) and isogenic mutants. MAC-T cells were infected with each of the four strains (WT, Δ*vraG*, *ΔhemB* and Δ*vraG*,*ΔhemB* [ΔΔ]) for 3h, then were incubated with lysostaphin for 12 h (A) or 24 h (B). MTT viability assays were then performed as described in materials and methods. The results are reported as percent viability relative to uninfected cells and are expressed as the mean with SD of three independent experiments done in triplicate. Statistical significance with the “Φ” symbols are compared to the WT, and the “*” symbols compare the indicated strains (Two-way ANOVA and Tukey's multiple comparisons test: * or Φ, P ≤ 0.05; **, P≤ 0.01; ***, P ≤ 0.001; ΦΦΦΦ, P ≤ 0.0001).

As expected, both SCV strains caused significantly less MAC-T cytotoxicity in this assay in contrast to that seen with the wild-type and Δ*vraG* strains: when compared to Δ*hemB*, the wild-type strain nearly reduced by half the viability of cells at 12 h (Fig 3A: wild-type, 25.4%; Δ*hemB*, 48.4%). This difference was still apparent at 24 h (Fig 3B: 16.25 *vs*. 34.55%, respectively), even if the bacterial load was 10 times higher for the Δ*hemB* mutant (Fig 1B). The MAC-T cells were more damaged by *ΔhemB* than by the double mutant Δ*vraG*Δ*hemB* but the difference was only significant at 24 h (P ≤ 0.01). The double mutant sustained epithelial cells viability 2.3 times more than the wild-type strain at 12 h (Fig 3A) and 2.7 times more at 24 h (Fig 3B) (P ≤ 0.0001 for both time points). Therefore, the greater intracellular persistence of both SCV strains compared to the wild-type and Δ*vraG* strains over time (Fig 2) was likely to be attributed to a lower toxicity of the SCVs for MAC-T cells (Fig 3).

Taken together, the results from the BMECs infection assays provide evidence of an additive effect of both *ΔhemB* and Δ*vraG* mutations for the attenuation of the wild-type strain; the *vraG* mutation mainly lowering the intracellular bacterial load and the *hemB* mutation creating the SCV phenotype that increases MAC-T cells viability.

### Δ*vraG*Δ*hemB* double mutant is strongly attenuated in a mouse IMI model and is efficiently cleared from mammary glands

**Fig 4:**
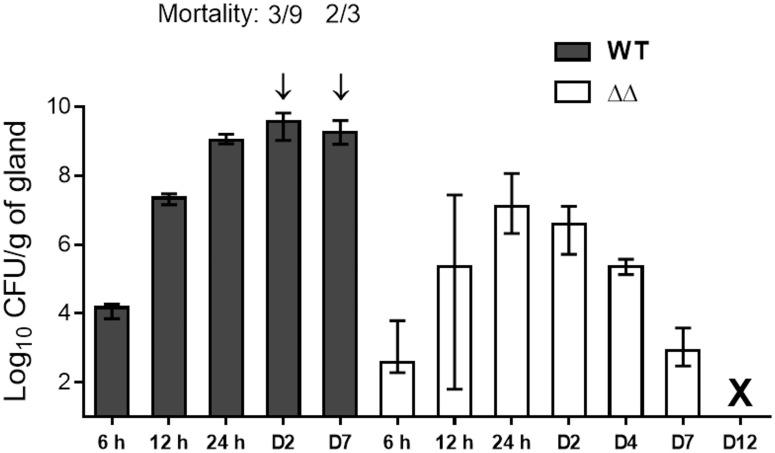
Murine IMIs with the parental (WT) and Δ*vraG*Δ*hemB* (ΔΔ) strains. Mice were infected as previously described and glands were harvested at the indicated hour (h) or day (D) after infection. Each column represents the median value of bacterial CFU counts for a group of glands, and ranges are indicated by bars. A minimum of six glands per group were used except for the WT strain at D7 (2 glands: only one mouse survived). Mortality of mice at specific time points is indicated by arrows. The X indicates the clearance of Δ*vraG*Δ*hemB* from glands (below the detection limit of 10 CFU/gland).

To attest the attenuation of Δ*vraG*Δ*hemB* in an *in vivo* model of infection, the virulence of the double mutant was evaluated and compared to the wild-type strain in a murine IMI model. For both strains, the exponential phase of infection took place mainly within the first 12 h post-infection, while the maximal bacterial burden was reached at 24 h for the double mutant and 48 h (day 2 [D2]) for the wild-type strain (Fig 4). At 24 h, the double mutant showed a reduction of 1.9 log10 in mean CFU/g of gland compared to the wild-type (P ≤ 0.05). Also after 24 h, the mutant bacterial burden showed a constant decline until complete bacterial clearance was reached at day 12 (shown by the asterisk on Fig 4). In contrast, the parental strain provoked severe invasive infections compared to the mutant, killing 3 of the 9 remaining mice at day 2 and 2 of 3 mice at day 7 (Fig 4; arrows) before glands could be harvested for those groups. Mice surviving the WT infection maintained high viable counts (9 log10 CFU/g of gland) at day 7, an approximate 5 log10 difference in bacterial burden compared to the double mutant. These results clearly demonstrate a markedly reduced capacity of the double mutant Δ*vraG*Δ*hemB* to multiply and survive in the mammary gland.

### Inflammatory response to Δ*vraG*Δ*hemB* and WT strains following IMI

**Fig 5:**
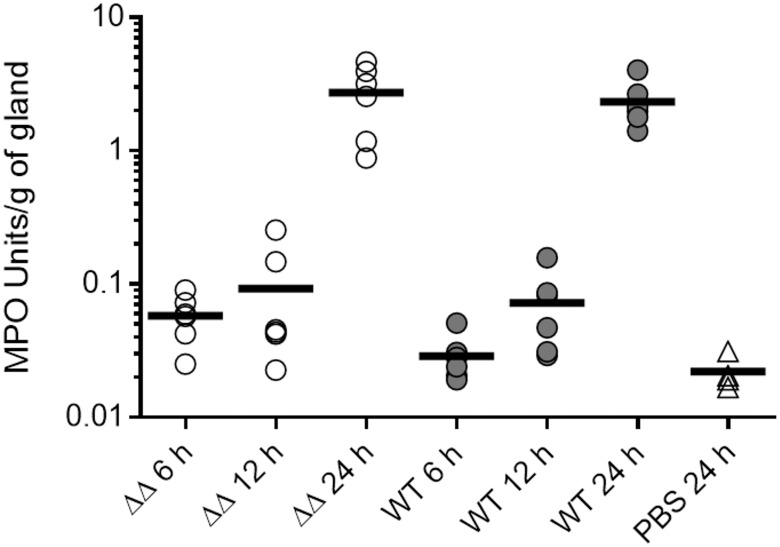
Double mutant Δ*vraG*Δ*hemB* (ΔΔ) stimulates neutrophil influx in mammary glands at levels comparable to *S*. *aureus* ATCC 29213 (WT) in the first 24 h of infection. Mice were infected as described in materials and methods, and a non-infected control group of mice received a sterile PBS injection (PBS). Glands were harvested at the indicated times, homogenized and kinetically assayed for MPO activity as described in materials and methods. Each dot represents MPO Units for one gland and is shown as a raw value adjusted by gram of gland. Means are represented by thick lines.

**Fig 6:**
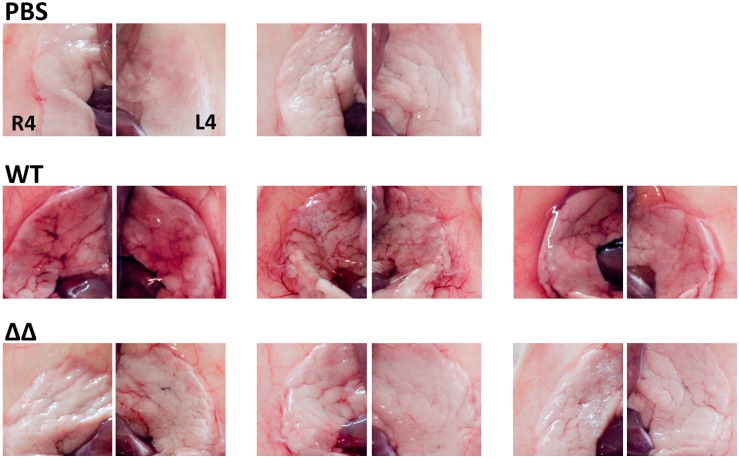
Visual inflammation of the large R4 and L4 mouse mammary glands 24 h after IMI with *S*. *aureus* ATCC 29213 (WT) and the double mutant Δ*vraG*Δ*hemB* (ΔΔ). Mice were infected as described in materials and methods, and the non-infected control group of mice received a sterile PBS injection (PBS). Pictures show the pairs of glands (R4, left, and L4, right) for each mouse in each group (PBS, n = 2 mice; WT, n = 3 mice; ΔΔ, n = 3 mice) that were harvested after 24 h.

To monitor the inflammatory response of the mice to infections with the wild-type and mutant strains, neutrophil infiltration in glands was evaluated by the MPO enzymatic activity in gland homogenates. MPO activity in biological samples has previously been correlated with the absolute number of neutrophils [47], and is hence an adequate representation of neutrophil infiltration. During the first hours after infection, neutrophil recruitment followed similar profiles for the double mutant and wild-type infected glands (Fig 5), with exponential intensification of apparent neutrophil infiltration from 12 h to 24 h post infection coinciding with bacterial growth albeit with a certain delay. We indeed previously showed that the absolute numbers of polymorphonuclear cells in relation to the bacterial load in mammary glands does not always peak at the same time [48]. No significant difference in MPO activity could be observed at 6, 12 and 24 h between glands infected by mutant and wild-type strains (Fig 5). This equivalence in apparent neutrophil infiltration did not however correlate with the visual observation of inflammation at 24 h, at which point the wild-type infection generated extensive redness of glands in comparison to the double mutant (photographs of Fig 6). In contrast, mutant infected glands were not visually altered at the macroscopic level compared to non-infected controls. The disparity between the visual assessment of inflammation and neutrophil infiltration results could be attributed to the differences in bacterial loads (Fig 4) and the cytotoxic activity of the wild-type strain (Fig 3). Hence, these results indicate that neutrophil recruitment in the glands infected by the double mutant ΔvraGΔ*hemB* strain was equivalent to that seen with the wild-type strain and that this was sufficient to allow a subsequent decline and clearance of the mutant bacterial loads.

### The inflammatory response of Δ*vraG*Δ*hemB*-infected glands goes back to normal levels with bacterial clearance

**Fig 7:**
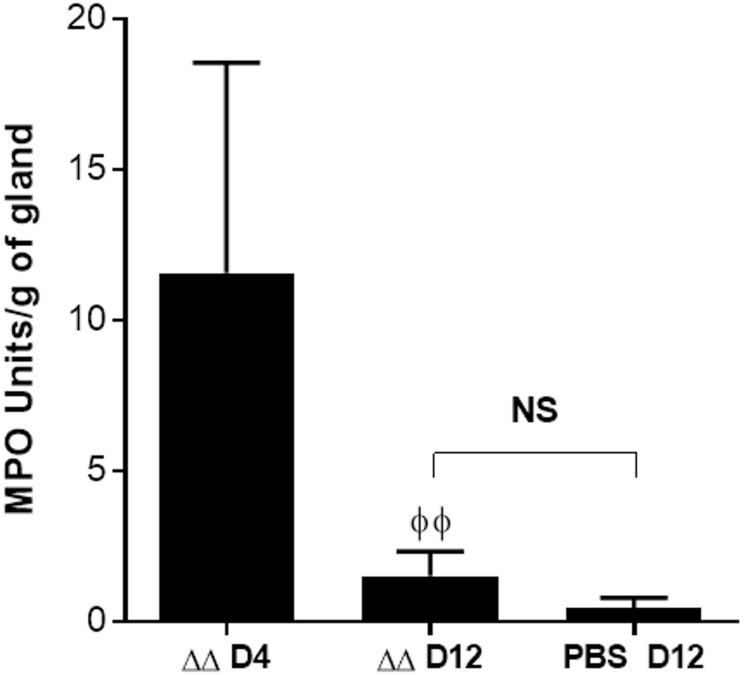
Neutrophil infiltration goes back to normal levels after clearance of the double mutant Δ*vraG*Δ*hemB* (ΔΔ) from the mammary glands. Mice were infected as described in materials and methods, and a non-infected control group of mice received a sterile PBS injection (PBS). Glands were harvested at the indicated times, homogenized and kinetically assayed for MPO activity as described in materials and methods. Columns represent means of MPO Units of a group of 6 glands (4 for the PBS control) adjusted by gram of gland, and error bars illustrate standard deviation. Statistical significance between the Day 4 and 12 groups post infection is shown by the “Φ” symbols. One-Way ANOVA and Tukey’s multiple comparison tests were used (ΦΦ, P≤ 0.01; NS, no significant difference between groups).

In order to attest strain safety, keeping in mind the possible use of the double mutant as a live-attenuated vaccine, and to confirm that this inflammatory response was not consequent to an inadmissible reactogenic strain, we continued monitoring of MPO activity in Δ*vraG*Δ*hemB*-infected glands 4 and 12 days after infection. The level of MPO activity was then compared to levels obtained for glands from non-infected mice. As illustrated in Fig 7, the apparent neutrophil presence in mutant infected glands was still high 4 days after infection, with MPO activity ranging from 8 to 21 Units/g of gland. The levels of MPO at this time point might be the direct consequence of the mammary gland involution, the process by which the lactating gland returns to a morphologically near pre-pregnant state. Indeed, involution is normally associated with neutrophil recruitment allowing phagocytosis of apoptotic cells during the remodelling of tissue [49]. However, later on, the MPO levels in the mutant infected glands went through a substantial decline between days 4 and 12, (P ≤ 0.01). MPO concentration was then considered to be back to normal levels at day 12, showing no significant difference with the non-infected glands.

### Immunizations with Δ*vraG*Δ*hemB* generate a strong humoral response against several *S*. *aureus* bovine IMI isolates

**Fig 8:**
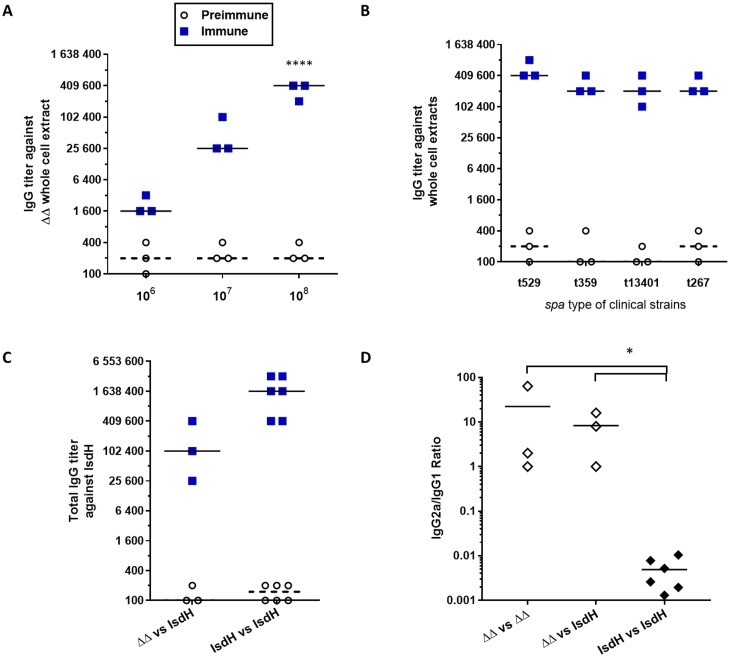
Immunization of mice with the live-attenuated double mutant (Δ720Δ*hemB*) induces a strong humoral response against *S*. *aureus* bovine mastitis isolates and against a specific cell-wall associated antigen (IsdH). Mice were immunized as previously described: serums were collected before priming immunization (preimmune, open circles) and ten days after the boost immunization (immune, blue squares). **A.** IgG titers rise with increasing immunization doses (10^6^, 10^7^, 10^8^ CFU) of the live-attenuated mutant Δ*vraG*Δ*hemB*: each dot represents the total IgG titer of one mouse against a Δ*vraG*Δ*hemB* whole cell extract. Medians are represented by thick lines for immune titers and dashed lines for preimmune titers. Titers were compared to their corresponding preimmune titers (Two-way ANOVA and Tukey’s multiple comparisons test: ****: P ≤ 0.0001). **B.** Immunization with the live-attenuated mutant Δ*vraG*Δ*hemB* confers high IgG titers against components that are shared by mastitis strains of commonly found *spa* types. Each dot represents the total IgG titer of one mouse against the whole cell extract of the indicated strain. Medians are represented by thick lines for immune titers and dashed lines for preimmune titers. All immune titers were compared to their corresponding preimmune titers (Two-way ANOVA and Tukey’s multiple comparisons test: P ≤ 0.0001 for all groups). **C.** Immunization with the live-attenuated mutant Δ*vraG*Δ*hemB* confers specific IgG titers against the cell-wall associated protein IsdH. Each dot represents the total IgG titer of one mouse against recombinant IsdH. Compared groups were immunized with the 10^8^ CFU of the live-attenuated Δ*vraG*Δ*hemB* (ΔΔ) or 25 μg of the purified recombinant IsdH protein (IsdH). **D.** IgG isotype ratios (IgG2a/IgG1) of mice immunized with the live-attenuated mutant Δ*vraG*Δ*hemB* (open diamonds) or immunized with the recombinant IsdH (black diamonds), against whole-cell extracts of strain Δ*vraG*Δ*hemB* (vs ΔΔ) or against the recombinant IsdH protein (vs IsdH). Each diamond represents the IgG2a/IgG1 titer ratio for one mouse. Medians are represented by thick lines (One-way ANOVA and Dunn’s multiple comparison test: *: P ≤ 0.05).

To confirm that immunization with the attenuated strain Δ*vraG*Δ*hemB* can indeed generate a strong immune response suitable for its use as a putative live vaccine against *S*. *aureus* IMIs, mice were immunized with different doses of the mutant and serum total IgGs were assayed by ELISA for detection of antigenic components present in whole-cell extracts of a variety of *S*. *aureus* bovine isolates. A specific detection of the staphylococcal iron-regulated IsdH protein was also attempted by ELISA. Doses of 10^6^, 10^7^ and 10^8^ CFUs, when administered subcutaneously in the neck, triggered no adverse effect such as modification of mice behavior, signs of inflammation, or necrosis at the immunization site throughout the immunization period. Additionally, immunizations using increasing quantities of the live double mutant Δ*vraG*Δ*hemB* yielded increasing titers of systemic IgG antibodies against its own whole cell extract (Fig 8A). The titers of the immune sera were significantly higher than those of the preimmune sera, demonstrating specificity of antibody production against the *S*. *aureus* antigens present in the live vaccine. Most importantly, increasing the doses of Δ*vra*GΔ*hemB* also generated a consequential rise of antibody titers against a variety *S*. *aureus* strains isolated from bovine mastitis, including strains from the major *spa* types found in Canada and elsewhere in the world (Fig 8B). Interestingly, it was also possible to generate specific IgGs against the cell wall-associated and iron-regulated protein IsdH as demonstrated in the ELISA using this protein as the antigen (Fig 8C). These results clearly show that (i) immunization with the double mutant can raise a specific immune response against *S*. *aureus*, and that (ii) the strain background (ATCC 29213) share sufficient common features with bovine mastitis strains so that the antibody response also strongly recognizes strains of major *spa* types. Additionally, the presence of IgG2a and IgG1 isotypes specific to IsdH, *i*.*e*., indicative of a Th1 and Th2 oriented immune response, respectively, was assayed for serums collected from mice immunized with the double mutant and compared to that obtained from mice immunized with the purifed IsdH protein. Significantly higher IgG2a/IgG1 titer ratios (*P* ≤ 0.05) were found for serums from mice immunized with the live-attenuated double mutant compared to the ratios obtained from mice vaccinated with the purified IsdH protein (Fig 8D).

## Discussion

The ability of *Staphylococcus aureus* to express multiple virulence factors permitting host colonization, tissue destruction, immune evasion, intracellular persistence and biofilm production makes it a very challenging pathogen to fight. Vaccines designed to prevent IMI in bovine mastitis therefore have to take into account the complexity of *S*. *aureus* pathogenesis as well as the diversity of strains capable of causing mastitis including strains with the SCV phenotype. SCVs are known to be somewhat attenuated but have intracellular abilities that allow persistence in the host without producing invasive infections [24]. In this study, we further attenuated the SCV phenotype to demonstrate that this phenotype could be used as a live attenuated vaccine.

One of our recent research endeavors has been to identify genes that are highly expressed by multiple *S*. *aureus* strains *in vivo*. The proteins encoded by these genes represent good targets as vaccination agents or in drug development as they are more likely to have an importance in virulence and, being expressed, to be efficiently targeted by the immune response. In a previous study, we used a DNA microarray approach to uncover *S*. *aureus* genes that were highly expressed by several strains in experimentally induced bovine IMIs [19]. The *S*. *aureus* operon *vraFG* (SACOL0718-720) was among several genes strongly expressed by *S*. *aureus* in the mammary gland environment [19].

The operon *vraFG* codes for an ABC transporter-like system with a role in resistance to antibiotics [32,50–52] and to several cationic antimicrobial peptides (CAMPs) such as indolicidin isolated from bovine neutrophils [30,53], human cathelicidin LL-37 [54] and Class I bacteriocins such as nisin A and nukacin ISK-1 [55]. Noteworthy, *vraFG* was shown not only to be under the regulation of the two-component regulatory system *graXRS*, but also to play an essential role by sensing the presence of CAMPs and signaling through *graS* to activate *graR*-dependent transcription, including its own transcript [30]. Besides, *vraFG* does not act as a detoxification module as previously believed [32], as it cannot confer resistance when produced on its own [30]. It was also reported that the expression of two key determinants, *mprF* and *dlt*, (needed for the modification of bacterial surface charged residues) is dependent upon *graXRS-vraFG*, and that these effectors are responsible for making the surface charge globally less negative [32], thus promoting resistance. When the sensing system or its effectors are altered, an increased susceptibility to vancomycin [32], daptomycin, polymyxin B [52] and several host defense CAMPs [56] is observed.

Our previous studies revealed that gene *vraFG* (SACOL0718-720) was up-regulated in both fresh milk *in vitro* and in milk recovered from infected cows. But of greater significance, this gene was shown to be a key factor in *S*. *aureus* virulence in cows, since a Δ*vraG* mutant was greatly attenuated in experimental bovine IMIs [19]. Consequently, this mutation was selected to further attenuate the SCV phenotype by generating the double mutant (Δ*vraG*Δ*hemB*) investigated in the present work.

This study is the first one, to our knowledge, to consider the use of classical respiratory deficient SCVs as the foundation of a non-virulent, genetically-defined attenuated vaccine for the delivery of *S*. *aureus* antigens. Live-attenuated strains of *S*. *aureus* have been of great interest for a long time and have been studied for immunization of cows since the ‘80s [57]. Some teams have managed to produce attenuation by chemical mutagenesis [58] in order to elicit high specific humoral response in cows, but unfortunately this caused only a weak reduction in shedding of bacteria, and no difference in the reduction of somatic cell counts (SCC) in milk when vaccinated groups were challenged. Besides, the genetic basis for the attenuation of this strain was still unknown, which may be a concern considering the necessity to obtain a stable and safe vaccine. In a different manner, transposon mutagenesis was used to generate an aromatic amino acid auxotrophic *aroA* mutant of *S*. *aureus* for testing in a mouse IMI model [59]. Both Th1 and Th2 responses were elicited, and a certain degree of protection was observed against homologous and heterologous *S*. *aureus*. The mutant was also demonstrated safe in leukopenic mice in a model of nasal colonization [60], but its immunogenicity in cows remains unknown.

In this study, genetic stabilization of the SCV phenotype (*i*.*e*., the *hemB* deletion) along with inactivation of an important effector of the resistance to cationic compounds (*i*.*e*., the *vraG* deletion) were able to generate an attenuated *S*. *aureus* strain that still exhibited a low transient internalization in epithelial cells. Since SCVs are expected to show a high capacity of invasion and intracellular persistence [23], the reduction we observed in post-invasion intracellular bacterial loads was attributed to the disruption of gene *vraG*. Since inappropriately high intracellular invasion and persistence might not be suited for a strain intended to be used as a live vaccine (even if low internalization in cells might help stimulating cell-mediated immunity), this second mutation was considered relevant for attenuation, especially in the SCV background.

More specifically; the lesser degree of internalization and intracellular persistence in BMECs observed for Δ*vraG* and especially for Δ*vraG*Δ*hemB*, as well as the total clearance of the latter mutant from glands in mice, suggested an additive deleterious effect of the two mutations. Because of their reduced membrane potential (ΔΨ), respiratory deficient SCVs (having an altered electron transport chain) are generally expected to be more resistant to cationic compounds or antibiotics that require membrane polarization for their mode of action [27,61–62]. However, other unknown mechanisms and factors can also lead to a decreased [63], or even a higher susceptibility of SCVs to such compounds, as previously shown with the frog-derived CAMP dermaseptin [64]. Also, electron transport SCVs have been shown to be more susceptible to oxidant damage caused to their membrane, because of their limited ability to generate a ΔΨ [65]. Therefore, it is likely that disruption of the *graXRS-vraFG* regulon via *vraG* mutation in the SCV background (*i*.*e*., Δ*vraG*Δ*hemB*) may be more deleterious than that seen with the normal phenotype (*i*.*e*., Δ*vraG*) because of the lack of membrane potential, which is required for active detoxification and reactive oxygen species (ROS) protection [66].

Another explanation for the strong attenuation seen for the double mutant is the possibility that *graXRS* and *vraFG* act as key regulators in the stress response of SCVs. The alternative transcription factor sigma B (SigB) is known to affect the expression of several genes encoding virulence factors and stress-response systems specific to SCVs [21]. This regulator has a permanent activity in *hemB* mutants [67] and was shown to play a role in biofilm production and in the intracellular persistence of SCVs [21]. VraFG may act in concert with SigB constant influence or possibly through another mechanism involving PhoU. PhoU is a global negative regulator of genes involved in central carbon metabolism and cytochrome expression and is therefore connected to the SCV phenotype [61]. In *S*. *aureus*, PhoU is important for resistance to CAMPs [68] and has been shown to regulate *dlt*, which is also under the control of *graXRS-vraFG*. Besides, GraSR has been linked to virulence and stress response pathways, which could help the SCV and normal phenotypes to persist in the host environment [69].

The low expression of invasive virulence factors such as hemolysin-α and other toxins associated with the reduced quorum-sensing activity of SCVs [39], probably resulted in the relatively low BMEC cytotoxicity of SCVs observed in this study. Nevertheless, the precise mechanisms by which non-SCV *S*. *aureus* strains kill epithelial cells are not completely understood and could be attributed to both induction of apoptotic pathways and/or pore-forming related lysis [23,27]. One of the prominent results of this study hinges on the high attenuation of virulence that was attained with the double mutant in the mouse IMI model. The parental strain was highly virulent and resulted in considerable mortality in this model, whereas a 5-log10 reduction in CFU/g of gland followed by total bacterial clearance from the glands was observed for the double mutant. The double mutant strain showed a good capacity to stimulate the recruitment of neutrophils in the gland and most importantly, this inflammatory response was not associated with tissue damage. Histopathological examinations of inoculated glands in future investigations will help to further support innocuity at the microscopic level.

This pro-inflammatory response was a first clear indicator of the potential of the double mutant strain as a live-attenuated vaccine. When administered through the subcutaneous route, the marked attenuation of the double mutant permitted the use of relatively high doses of live bacteria to immunize mice, without provoking any sign of local inflammation or adverse effect. At the same time, this immunization allowed to trigger a broad systemic response that translated in high IgG titers against whole *S*. *aureus* cell components. This humoral response was also broad enough to react against several bovine mastitis isolates represented by the most prevalent *S*. *aureus spa* types found in Canadian dairy herds [13] and elsewhere in the world [34]. Furthermore, the response was found to include significant IgG titers against the staphylococcal iron-regulated and cell-wall associated IsdH antigen. IgG isotypes produced against this antigen also allowed to demonstrate a more balanced Th1 and Th2 response as compared to that obtained when immunizing with the purified IsdH antigen. This feature might help to improve protection against *S*. *aureus*, for which control is increasingly thought to require cell-mediated immunity [15–18]. Noteworthy, although this proof of concept demonstrated that the double mutant genetic background (ATCC 29213) share many common features with bovine mastitis strains, such mutations (*i*.*e*., Δ*vraG*Δ*hemB*) and attenuation can be created in any desired background if one wishes to cover specific types of strains. The demonstration of protection elicited by such a vaccine against experimental IMI in mice, and then in cows, will need to be examined in future work, along with investigations on the best possible route of administration. The use of cows is clearly important for future studies; nevertheless, we have recently shown that results from our mouse model of IMI [70] can translate very well to that obtained in cows [71].

As a final note, the administration route of such vaccines might undeniably influence the qualitative properties of immune response and efficacy of protection. On this matter, it was previously reported that intramammary but not intraperitoneal application of live temperature-sensitive *S*. *aureus* could stimulate murine mucosal responses against a challenge with a homologous virulent strain [72]. A different study was conducted by using formalin-killed whole cells of planktonic and biofilm *S*. *aureus* to immunize mice [73]. It was shown that the biofilm vaccine performed better in immunogenicity and protection when administered by the intramammary route, despite the fact that the planktonic subcutaneous vaccine triggered a significantly higher humoral response. In more recent work, the same team reported that subcutaneous immunizations with staphylococcal protein A could elicit higher humoral responses against the antigen, but that the response was more balanced (humoral and cellular) when administered by intramammary injections [74]. However, this subunit vaccine failed to protect immunized mice challenged (IMI) with a strong biofilm-producing and encapsulated *S*. *aureus* strain, regardless of the route of immunization. In this manner, the route of administration of our genetically defined live-attenuated vaccine will definitively impact the level of its protective efficacy, and additional practical aspects will need to be considered (*e*.*g*., subcutaneous administration *vs*. intramammary perfusion into four quarters for a whole herd) in upcoming studies.
